# Supplementary material for: Stigma towards mental illness and help-seeking behaviors among adult and child psychiatrists in Hungary: A cross-sectional study
Source: PLoS One. 2022 Jun 10;17(6):e0269802. doi: 10.1371/journal.pone.0269802 (PMC9187077; doi:10.1371/journal.pone.0269802)
Supplement: S1 Questionnaire — (DOCX) [file pone.0269802.s001.docx]

**Supporting Information**

**Questionnaire**

1. Have you ever experienced any negative discrimination for working as a psychiatrist?

Yes

No

1. What is your gender?

Male

Female

Prefer not to answer

1. How old are you?

24-35

36-45

46-55

56-65

66-75

76<

1. Which field do you work in?

General adult psychiatry

Child and adolescent psychiatry

1. What is your current status?

Trainee

Specialist

1. How many years of experience do you have in psychiatry?

0-5 years

5-10 years

11-20 years

21-30 years

31-40 years

more than 40 years

1. How many percent of your working hours do your spend with patients?

0-25%

25-75%

>76%

1. Where do you work?

Urban area

Rural area

1. If urban area, what is the population?

City above 3 million inhabitants

City between 1-3 million inhabitants

City between 500.000 -1 million inhabitants

City less then 500.000 inhabitants.

1. What kind of practice do you work at in the majority of your working hours?

Inpatient hospital

Psychiatric outpatient service

Other outpatient service where psychiatric patients are also treated

Day-care service

Exclusively private practice

I do not work in patient care

Other

1. Do you do currently psychotherapy?

Yes

No

1. Do you have any friends or family members who are dealing with mental illness?

Yes

No

I do not know

1. Have you ever sought help for your own mental health problems?

Yes

No

Prefer not to answer

1. Have you ever been medically treated for any psychiatric problems?

Yes

No

Prefer not to answer

1. Have you ever attended psychotherapy for your own mental health problems?

Yes

No

Prefer not to answer

1. Have you ever attended any kind of psychotherapy for professional purposes (work, training, education)?

Yes, group sessions

Yes, individual sessions

Yes, booth group and individual sessions

None of the above

1. How many hours have you spent in psychotherapy (for professional and/or personal purposes)?
2. Are you currently in psychotherapy for any reason?

Yes

No

1. Are you open to participating in case discussion groups, supervision, or Balint-groups?

Yes

No

1. Is it an option for you to participate in case discussion groups, supervision, or Balint-groups?

Yes

No

1. Have your patients ever experienced any stigmatizing attitude from other mental health professionals?

Yes

No

Please read each of the following statements and rate the extent to which it describes your feelings about people with mental illness.

1. I am more comfortable helping a person who has a physical illness than I am helping a person who has a mental illness.

strongly agree

agree

neither agree nor disagree

disagree

strongly disagree

1. If a colleague with whom I work told me they had a managed mental illness, I would be as willing to work with him/her.

strongly agree

agree

neither agree nor disagree

disagree

strongly disagree

1. If I were under treatment for a mental illness I would not disclose this to any of my colleagues.

strongly agree

agree

neither agree nor disagree

disagree

strongly disagree

1. I would see myself as weak if I had a mental illness and could not fix it myself.

strongly agree

agree

neither agree nor disagree

disagree

strongly disagree

1. I would be reluctant to seek help if I had a mental illness.

strongly agree

agree

neither agree nor disagree

disagree

strongly disagree

1. Employers should hire a person with a managed mental illness if he/she is the best person for the job.

strongly agree

agree

neither agree nor disagree

disagree

strongly disagree

1. I would still go to a physician if I knew that the physician had been treated for a mental illness.

strongly agree

agree

neither agree nor disagree

disagree

strongly disagree

1. If I had a mental illness, I would tell my friends.

strongly agree

agree

neither agree nor disagree

disagree

strongly disagree

1. Despite my professional beliefs, I have negative reactions towards people who have mental illness.

strongly agree

agree

neither agree nor disagree

disagree

strongly disagree

1. There is little I can do to help people with mental illness.

strongly agree

agree

neither agree nor disagree

disagree

strongly disagree

1. More than half of people with mental illness don’t try hard enough to get better.

strongly agree

agree

neither agree nor disagree

disagree

strongly disagree

1. I would not want a person with a mental illness, even if it were appropriately managed, to work with children.

strongly agree

agree

neither agree nor disagree

disagree

strongly disagree

1. Health care providers do not need to be advocates for people with mental illness

strongly agree

agree

neither agree nor disagree

disagree

strongly disagree

1. I would not mind if a person with a mental illness lived next door to me.

strongly agree

agree

neither agree nor disagree

disagree

strongly disagree

1. I struggle to feel compassion for a person with a mental illness.

strongly agree

agree

neither agree nor disagree

disagree

strongly disagree

1. In your opinion, to what extent do your colleagues (psychiatrists) show a stigmatizing attitude towards patients with mental illness?

not at all

to small extent

to some extent

to great extent
