# Supplementary material for: Stigma towards mental illness and help-seeking behaviors among adult and child psychiatrists in Hungary: A cross-sectional study
Source: PLoS One. 2022 Jun 10;17(6):e0269802. doi: 10.1371/journal.pone.0269802 (PMC9187077; doi:10.1371/journal.pone.0269802)
Supplement: Attachment — Submitted filename: Review Apr 2022.docx [file pone.0269802.s002.docx]

**Stigma towards mental illness and help seeking behaviours amount adult and child psychiatrist in Hungary: across sectional study**

Thank you for the opportunity to review. Overall this is an interesting paper with new information to add to the field. I have the following questions and comments/suggestions for improvement for the authors’ consideration:

1. The notion of stigma is complex and the semantics can be confusing. Suggest the authors elaborate more clearly what they mean by stigma in a number of places throughout the Introduction. For example, page 4 first paragraph last sentence: “… the support of professionals might play and essential role in mitigating stigma since higher levels of burnout were associated with more negative attitudes towards patients”. It is unclear what are the authors mean - support for professionals will mitigate having stigmatizing attitudes, or mitigating stigma in their patients, or in society in general. Page 3, last sentence -…”demand for personal accomplishment and burn out or predictors of stigma…” Again this stigma is unclear in what the authors are referring to. Please review carefully throughout the introduction.
2. Page 3, it will be helpful to explain the Weak-not-sick scale for the readership.
3. A broader review of how to support mental health workers will be helpful, rather than providing two choices such as case discussion or a Balint group. It’s as if these are already well accepted and only choices. If there are evidence and literature to support their effectiveness please provide such.
4. Page 4, Methods. The research group contacted 60 child psychiatric inpatient services and 69 outpatient services with many other efforts. It will be helpful to estimate what are the total number of potential recipients here (only later mentioned in the Results ), and the percentage that the 238 respondents represent. The response rates seem very low, and more analysis and explanations of the rate of response would be helpful. This should be a limitation in the discussion as well.
5. Page 4, last paragraph that talks about the Hungarian version of the OMSHC seem out of place in describing the process of translation and concept checking, after citing the psychometric properties and the test retest reliability of the Hungarian version already in the preceding paragraph. This could be either incorporated in introducing this Hungarian version or be omitted.
6. Also it is unclear what do the authors mean by “a good concurrent validity was calculated with a mental illness clinicians attitude- 4 scale. More explanation is needed.
7. Under statistics, there is no mention of multi test corrections since this is a large item by item analyses in correlations and regressions. A statistics expert reviewer may be needed.
8. There are a number of small grammatical errors that could be checked. For examples: Page 14 second last paragraph “practitioner’s personal affection as well” is poor choice of words. Missing a verb in the sentence about nursing students from Singapore, and they were “one fifth” on top of page 15, etc.
9. In the Discussion I wonder whether the common phenomenon of people wanting to provide socially approved answers, particularly given that the survey is for medical professionals should be considered as a factor for discussion. The authors do talk confidently about “unequivocal demonstration of a positive attitude.” This is a worthwhile in any survey to consider, even if done anonymously.
10. One would hope for more in-depth reflections and discussions on why certain findings are the way they are, in addition to the numerical comparisons with other settings such as those in Singapore or Brazil or Bahrain. These comparisons themselves do not give rise to understanding beyond a cross cultural difference that the authors are bringing to light. The explanations about why a group discussion helps to lower stigma is on the right track, but it could be further illuminated on why such contact or discussion would lead to lowering of stigma.
11. Are there theories within mental health stigma that the authors could invoke.? It would be helpful to find a theory that the authors would like to anchor some of their findings rather than relying on statistical correlational results as an explanation in and of itself.
12. Last paragraph, page 16, interesting to highlight that psychiatrist who have experienced discrimination have more distance from their patients. Do the authors have some understanding in theory rather than stating that it’s an existing issue that needs to be further addressed?
13. Limitations: the very low response rate, the statistical corrections needed, why were some people not available, what are the reasons behind her low response and why is the sampling method more likely to reach and get responses from the younger individuals, what biases might have these selective samples introduced to the results should all be explored and discussed.
14. Strength of the paper is, as the authors also concluded, that there is a limited number of studies on psychiatrists and issues related to stigma. And it’s a solid study that can be further improved.
